# The Book World of Medicine and Science

**Published:** 1898-11-26

**Authors:** 


### The Book World of Medicine and Science.

Health Loss and Gain: A Plea for the More Definite and Extended Practice of Preventive Medicine. By M. A. Chreiman. (London: The Rebman Publishing Company. 1898. Pp. 226. Price 3s. net.)

To any medical man desiring half an hour's innocent laughter we can cordially recommend this amusing advertisement. There is no need to read it through from cover to cover; open it where he may he cannot fail to be diverted. Style, orthography, and grammar are alike original, and extraordinarily lurid effects are often produced by the introduction of misspelt medical words in inappropriate places. The authoress—for the internal evidence as to sex is indubitable—couches most of her remarks in the second person, addressing them to "You, the leaders of medicine." It appears that many of the most eminent members of our profession are in the habit of confiding their secrets to her, but she is kind enough to spare us their names while revealing the unprofessional nature of their conduct. We cannot but admire her self-restraint, which perhaps it might have been as well to have extended to the accounts of the cures which she herself has effected by her own system of remedial exercises under Royal and noble patronage (see pp. 165, 166). These accounts form, of course, the kernel of the work; the number and variety of the husks in which it is enclosed can only be adequately indicated by quoting the titles of the eleven sections of which it consists. These are in order, Preface; Protest; Apology, Explanation, and Prayer; Prevention and its Cost; Verities and Credulities; Respiration; Confirmation; Queries; Results of the Practice of Preventive Medicine; A Few Items of Personal Experience; and Church and State. Surely the most omnivorous reader could demand no more, and as the authoress usually attaches to these high-sounding designations meanings quite other than are commonly accepted, there is always the additional pleasure of unexpectedness. The book is excellently got up in the blue covers affected by the publishers.

Handbook of Obstetric Nursing. By Francis W. N. Haultain, M.D., F.R.C.P.Ed., and James Haig Ferguson, M.D., F.R.C.P.Ed., M.R.C.S.Eng. Third edition, revised and enlarged, with coloured plate and 37 engravings. (Edinburgh and London: Young J. Pentland.)

A great deal of care has evidently been expended upon the production of this book, which is, on the whole, a satisfactory presentment of the subject as taught in the Edinburgh school. It is, perhaps, not very apparent what is meant by obstetric nursing, for in some parts of the book it seems as if midwives are being addressed, while in others monthly nurses appear to be in the authors' minds. The fact is that here, as elsewhere, great confusion is introduced into the whole subject by the application of the term "nurse" to a person who is not a nurse, but a midwife. If we were inclined to be critical we should suggest that the writers seem to have found it difficult to attune their teaching to the degree of knowledge, or rather of ignorance, which is to be expected in pupils who, as is stated in the preface, "have no special previous training," and whose "knowledge necessary to gain the diploma must be acquired in three months." In one sense there is too much detail, in another sense too little. When the nurse is told that in preparing herself for attendance on a case "washing the hands with soap and water and turpentine and a new nail-brush is one of the best means of thoroughly cleansing them before dipping them in the antiseptic solution," we feel conscious of a vagueness of direction which is not altogether satisfactory, nor do we quite like the idea of the "nurse" merely "dipping" and "rinsing" her hands in antiseptics, seeing that the duties which she is to undertake involve all the internal examinations and manipulations which are usually undertaken by the practitioner. Then we cannot feel quite comfortable about women who are to receive a "diploma" after three months' training being taught that in the treatment of abortion opium should be given "either in the form of a morphia suppository, ½-grain, introduced into the rectum, or 15 drops of chlorodyne by the mouth, to be repeated at intervals of four hours," nor at their being told that if abortion be inevitable "efforts must be made to clear out the uterus as speedily as possible," and that in all such cases "a teaspoonful of the liquid extract of ergot should first be given, and repeated at intervals of two hours—this drug having been proved to have a most pronounced effect in strengthening uterine contractions." If it be said in excuse that, at the end of the chapter, it is directed that in all cases of abortion medical advice should be procured "if possible," we would point out, on the other hand, that earlier on it is stated that certain things should be done "if a doctor is also in attendance," showing that what is in the author's mind is that this will not always be the case, and that the directions given are such as may properly be followed by these "nurses." There are various points, both in regard to antiseptic methods and to the after treatment of the patient, as to which many good obstetricians might perhaps differ from the authors. Still, this is a handsome and well got up little work which no doubt well represents the school from which it springs.

A Pocket Dictionary of Hygiene. By C. T. Kingzett, F.I.C., and D. Homfrey, B.Sc. (London: Baillière, Tindall, and Cox. 1898. Pp. 103, 24mo. Price 2s. 6d.)

This little book is something more—or less—than a dictionary of hygiene. It gives, under perhaps a couple of hundred alphabetically arranged headings, condensed information on such various subjects as algæ, alcohol, bioplasm, filters, and slaughter-houses. For those who care to carry their knowledge of hygiene in the waistcoat pocket this is the very thing. It may be noted that the authors display a consistent prejudice in favour of one particular germicide, to the exclusion, indeed, of mention of all others. One might almost say, on putting the book down, that the object with which it is written is embodied in Disraeli's famous epigram, "Sanitas sanitatum omnia *Sanitas.*"

